# Initially High Correlation between Air Pollution and COVID-19 Mortality Declined to Zero as the Pandemic Progressed: There Is No Evidence for a Causal Link between Air Pollution and COVID-19 Vulnerability

**DOI:** 10.3390/ijerph191610000

**Published:** 2022-08-13

**Authors:** Brandon Michael Taylor, Michael Ash, Lawrence Peter King

**Affiliations:** Economics Department, University of Massachusetts Amherst, Amherst, MA 01003, USA

**Keywords:** COVID-19, PM_2.5_, air pollution, mortality, epidemiology, multiple regression

## Abstract

Wu et al. found a strong positive association between cumulative daily county-level COVID-19 mortality and long-term average PM_2.5_ concentrations for data up until September 2020. We replicated the results of Wu et al. and extended the analysis up until May 2022. The association between PM_2.5_ concentration and cumulative COVID-19 mortality fell sharply after September 2020. Using the data available from Wu et al.’s “updated_data” branch up until May 2022, we found that the effect of a 1 μg/m^3^ increase in PM_2.5_ was associated with only a +0.603% mortality difference. The 95% CI of this difference was between −0.560% and +1.78%, narrow bounds that include zero, with the upper bound far below the Wu et al. estimate. Short-term trends in the initial spread of COVID-19, not a long-term epidemiologic association, caused an early correlation between air pollution and COVID-19 mortality.

## 1. Introduction

It is of critical importance for public health to understand the correlation between vulnerability to COVID-19 infection and mortality. Early studies showed a high correlation between air pollution and a host of social vulnerabilities. We decided to replicate an early study of this relationship to further explore the study’s implications. However, after we replicated the study, we found that the correlation between air pollution and COVID-19 mortality appears to have been happenstance based on the early period of the pandemic. Other authors could consider whether the correlation of COVID-19 mortality with other social vulnerabilities has persisted throughout the pandemic.

Based on the early outbreak of COVID-19 cases in Northern Italy, Conticini et al. [1] first suggested that higher air pollution levels could increase COVID-19 mortality rates. Since then, many authors have analyzed this hypothesis. Brandt et al. stressed the environmental justice implications of this hypothesis: “lower income communities of color are more likely to have historical exposures to higher levels of air pollution. This chronic exposure is thought to worsen underlying diseases, including many that represent risk factors for severe COVID-19” [2]. In the Netherlands, Cole et al. found that the “relationship between COVID-19 and air pollution withstands a number of sensitivity and robustness exercises including instrumenting pollution to mitigate potential endogeneity in the measurement of pollution and modeling spatial spillovers” [3]. In France, Tchicaya et al. found “significant associations between the COVID-19 mortality rate and long-term exposure to air pollution and temperature” that “tended to decrease with the…massive spread of the disease across the entire country” [4]. In Queens, Adhikari and Yin found “a significant negative association among PM_2.5_ and new daily confirmed COVID-19 cases” and that “daily maximum eight-hour ozone concentration” is positively associated with “new confirmed cases” but not “new deaths” [5]. Milicevic et al. modeled COVID-19 as an epidemic and found that “a relative change in R0 [the basic reproduction number], with variations in pollution levels observed in the USA, is typically ~30%” [6]. Bourdrel et al. [7] suggested potential biological pathways but concluded that establishing a direct biological mechanism would be necessary. See also Appendix A, where we discuss additional literature.

**Figure 1:**
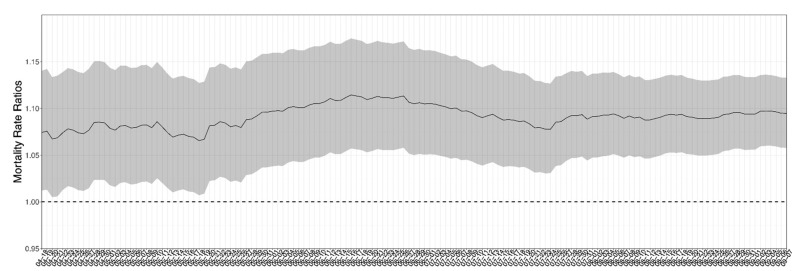
From Wu et al., original caption: “Figure S3: Daily COVID-19 mortality rate ratios (MRR) per 1 μg/m^3^ increase in PM_2.5_ and 95% CI”, with “unpublished updated results until 7 September 2020” [9]. Unfortunately, because we copied the original figure, we cannot change the crowded date labels in the *x*-axis. See the corresponding axis in Figure 2, which is less crowded.

Wu et al. [8,9] analyzed the relationship between air pollution and COVID-19 mortality for United States counties, using the cumulative death rate from the start of the pandemic (roughly January 2020, although no one knows for certain when the first person in the US contracted COVID-19) through 18 June 2020 as the dependent variable. Wu et al. found that one additional μg/m^3^ in the long-term average PM_2.5_ was associated with an 11% higher COVID-19 mortality rate (95% CI: 6% to 17%). Wu et al. used the average county-level PM_2.5_ concentration from 2000 to 2016 (a 17-year average) as the predictive variable. The US EPA standard for PM_2.5_ is 12.0 μg/m^3^, and at 609 monitored sites in 2020, the average value was 8.0 with 10th and 90th percentile average values of 5.6 and 10.8 across sites [10]. The association was statistically and practically significant and stable throughout both the published study period (from 18 April 2002 to 18 June 2020) and in a web-published extension, through 7 September 2020. We copied Figure 1 directly from the web-based extension [8]. Wu et al. showed (on the vertical axis) that the mortality rate ratio was associated with one additional μg/m^3^ of long-term average PM_2.5_ exposure using a negative binomial regression at the county level for cumulative mortality for each day of the pandemic (on the horizontal axis) through September 2020.

Take an example from the middle of the time-period to illustrate the cumulative mortality rates. The value of 1.10 for early June 2020 indicated that counties with one additional μg/m^3^ of long-term average PM_2.5_ exposure had a 10% higher mortality rate (all COVID-19 deaths from April 2020 to June 2020 divided by the pre-pandemic population). The mortality rate ratio from the regression peaked around 1.11 in mid-June 2020 and remained just under 1.10 in September 2020. Wu et al. found the association of air pollution with COVID-19 mortality while holding a wide range of explanatory variables associated with social vulnerability constant, including racial/ethnic composition, poverty, education, age structure, temperature, relative humidity, available hospital beds, population density, obesity, and smoking, by controlling for these variables.

## 2. Results

**Figure 2:**
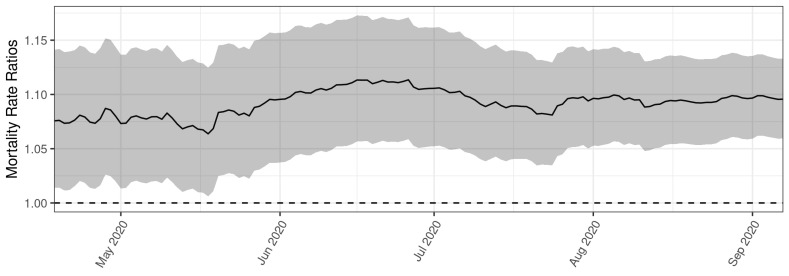
Our replication of Figure S3 from Wu et al. [9]. For each date, we reported the cumulative to-date mortality rate ratio associated with one additional μg/m^3^ of average long-term PM_2.5_ exposure at the county level for April through September 2020. We also shaded the 95% confidence interval of these ratios. We displayed date labels that are less crowded than the date labels in Figure 1 (above) to help readers interpret the figure.

We replicated Wu et al.’s results exactly. Figure 2 is our replication of Figure 1.

Our results matched the results of Wu et al. exactly. Thus, we successfully replicated the method and the finding of a stable, positive association between air pollution and COVID-19 mortality over the period analyzed by Wu et al.: from April to September 2020.

**Figure 3:**
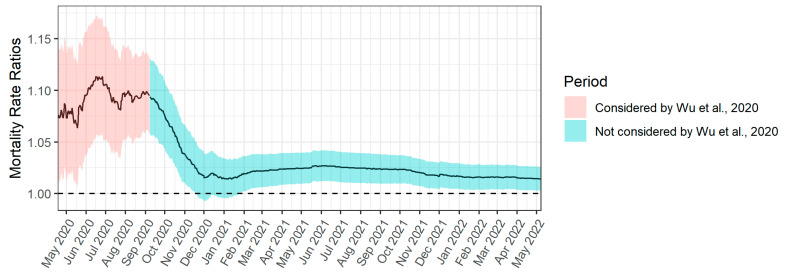
Extension of Figure 2 through May 2022. For each date, we reported the cumulative to-date mortality rate ratio associated with one additional μg/m^3^ of average long-term PM_2.5_ at the county level for April 2020 through May 2022 [8]. We also shaded the 95% confidence interval of these ratios.

We then extended the analysis to determine if the relationship between air pollution and COVID-19 mortality persists after the study period in Wu et al. In Figure 3, we applied the same method as in Figure 2. When we extended this analysis, we used the same average county-level PM_2.5_ concentrations from 2000 to 2016 that Wu et al. used. We also used the same array of potential confounding variables as Wu et al. We purposely used the exact same methods except that we used a longer time frame to estimate COVID-19 mortality, to show that only this change affected the results. As we analyzed the data cumulatively from the onset of the pandemic through each date on the horizontal axis, Figure 2, covering April through September 2020, appeared exactly as compressed as the first 7 months shown in Figure 3, which covered April 2020 through May 2022.

Each value is the mortality rate ratio for cumulative county mortality associated with a one μg/m^3^ increase in PM_2.5_ exposure.

The most striking feature of Figure 3 is the sharp decline in the association between PM_2.5_ air pollution and COVID-19 mortality after September 2020. The mortality rate ratio declined sharply and never again rose above 1.03. It is worth noting that the extremely deadly second wave of US COVID-19 coincides closely with the decrease in the relationship between COVID-19 mortality and air pollution. Additionally, vaccines only became available in early 2021, so vaccination cannot explain the decline that began in September. See Appendix B and Appendix C for further information.

The disappearance of the association between air pollution and COVID-19 mortality suggests that the association was particular to one phase (the first phase) of the pandemic and does not represent an important causal relationship between air pollution and COVID-19 mortality. Wu et al. were careful not to use causal language in their analysis. However, the Harvard School of Public Health did use causal language in their press release: “more evidence of causal link between air pollution and early death” [11].

**Figure 4:**
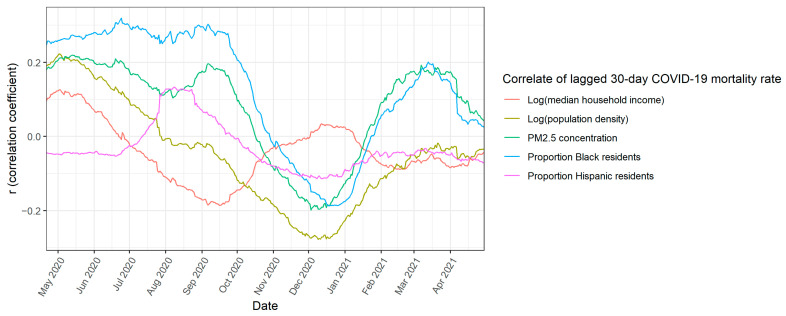
Simple correlations over the first year of the pandemic between demographic variables and the COVID-19 mortality rate within the previous 30 days.

Though Wu et al. used the best available data at the time, they found a short-term trend in the spread of COVID-19, not a long-term trend in COVID-19 mortality. To clarify, we are not referring to short-term and long-term exposure to air pollution. In Figure 4, we plotted the simple Pearson correlation coefficients (r, not R^2^) over the first year of the pandemic between several important demographic variables and the COVID-19 mortality rate for the previous 30 days. We used only the data from the previous month to separate short-term trends in the spread of COVID-19 from long-term trends in COVID-19 mortality. COVID-19 deaths increased in counties at uneven rates. For example, COVID-19 deaths increased disproportionately the fastest in Hispanic communities in early August. Between September 2020 and December 2020, the correlations among all five demographic variables, including PM_2.5_, changed significantly as COVID-19 spread into new communities.

None of these correlations are causally related to long-term COVID-19 mortality. Even though these are short-term relationships between demographic variables and the spread of COVID-19, the relationships are neither fair nor accidental. For example, it is certainly neither fair nor accidental that COVID-19 affected Black communities disproportionately more at the beginning of the pandemic: Black Americans are disproportionately more likely to live in urban areas, which were the locations where COVID-19 first spread. Moreover, in addition to short-term relationships between these variables and the spread of COVID-19, there may be additional long-term relationships between these variables and COVID-19 mortality. Nevertheless, the relationships outlined above represent short-term trends in the spread of COVID-19, not long-term trends in COVID-19 mortality.

## 3. Discussion

The association between COVID-19 mortality and air pollution was limited to the earliest phase and, we conclude, to the particular timing and location of the early COVID-19 outbreak. This is despite social inequalities exacerbating the toll of the pandemic, and the long-term inequality such as the differential exposure of vulnerable populations to air pollution (Zwickl et al., 2014).

Regardless of the specific reason for the short-term relationship between the spread of COVID-19 and PM_2.5_ concentrations, there is no long-term relationship between COVID-19 mortality and PM_2.5_ concentrations. Perhaps Wu et al. omitted an important variable, and this caused them to find a short-term relationship between the spread of COVID-19 and PM_2.5_ concentrations through September 2020. For example, imagine that long-distance travelers spread COVID-19 early during the pandemic. Long-distance travelers can also directly emit PM_2.5_ from their vehicles. Wu et al. might have falsely attributed unexplained differences in COVID-19 mortality to PM_2.5_ concentrations instead. When COVID-19 spread into counties with fewer long-distance travelers, these unexplained differences and the effect Wu et al. estimated decreased. However, we have not found any evidence for this explanation. Regardless of the specific reason for the short-term relationship between the spread of COVID-19 and PM_2.5_ concentrations, there is no long-term relationship between COVID-19 mortality and PM_2.5_ concentrations.

One could view these results as part of the so-called “replication crisis”. Ioannidis argued that “most published research findings are false” and that “a research finding is less likely to be true when the studies conducted in a field are smaller; when effect sizes are smaller; when there is a greater number and lesser preselection of tested relationships; where there is greater flexibility in designs, definitions, outcomes, and analytical modes; when there is greater financial and other interest and prejudice; and when more teams are involved in a scientific field in chase of statistical significance” [12].

Perhaps instead of viewing this as a replication “crisis”, we should view this as a replication “challenge”, for researchers to replicate more studies on new populations and for academic institutions and publishers to reward research that is not necessarily “first out of the gate”.

Because we used the same methodology as Wu et al., our study has the same limitations as theirs. An important and inherent limitation of ecological regression is that authors cannot guarantee that they have included all potential confounding variables. Wu et al. suggested that future authors should “quantify and correct for ecological bias and measurement error”, use “reproducible methods for causal inference”, and quantify “measured and unmeasured confounding bias”. Additionally, multiple regression is an inaccurate tool for modeling the spread of COVID-19 because COVID-19 infections are not confined by space and time. At the time that the article by Wu et al. was written, obtaining enough data for a diffusion model might have been difficult. However, as time passes, one can obtain more and more data on the spread of COVID-19, and some authors have indeed studied the relationship between PM_2.5_ and COVID-19 by modeling COVID-19 as a pandemic [6,13]. Ideally, Wu et al. would have considered mortality rates per COVID-19 case separately from the total number of COVID-19 cases. However, although Johns Hopkins reports the number of confirmed COVID-19 cases by county, subjects chose to test themselves, and this likely strongly biased these estimates. For example, a subject might be more likely to test themself for COVID-19 if the subject has symptoms of COVID-19 or is at a high risk for contracting COVID-19.

In the future, authors could consider how the duration of air-pollution exposure might (or might not) influence COVID-19 mortality rates. Wu et al. averaged PM_2.5_ concentrations over a long time-period prior to the pandemic, during and after which many people may have moved. This average may be a good indicator of long-term exposure, but not of exposure during the pandemic. When researchers estimate the effects of air pollution on all-cause mortality, they generally find that all-cause mortality increases more when exposure times are longer [14]. Some authors have found a similar result for the effect on COVID-19 mortality [15]. However, if the effect of long-term exposure is insignificant, the effect of short-term exposure is likely smaller and, thus, also insignificant. Even so, reducing air pollution would reduce the all-cause mortality rates of people exposed to air-pollution over both short and long durations [14].

## 4. Conclusions

In conclusion, while we observed a strong positive relationship between PM_2.5_ concentrations and COVID-19 mortality rates early during the pandemic, evidence for a causal relationship is limited. While data through September 2020 suggest that higher air pollution increased COVID-19 death rates, the same methods applied to data throughout mid-2022 showed little association between air pollution and COVID-19 mortality rates. Short-term trends in the initial spread of COVID-19, not a long-term epidemiologic association, caused an early correlation between air pollution and COVID-19 mortality.

## Figures and Tables

**Figure A1:**
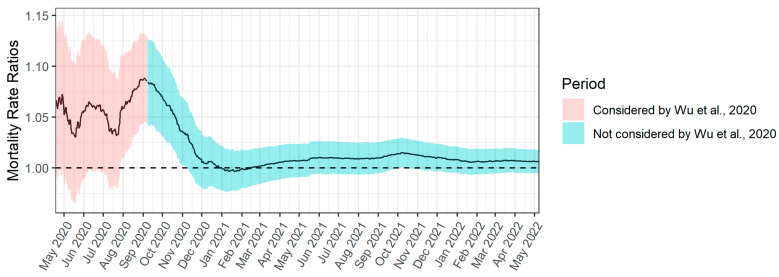
We used the data that Wu et al. 2020 [8] stored in the “updated_data” branch of their GitHub repository to recalculate Figure 3. For each date, we reported the cumulative to-date mortality rate ratio associated with one additional μg/m^3^ higher average long-term PM_2.5_ concentration at the county level for April 2020 through May 2022. We also shaded the 95% confidence intervals of these ratios.

**Figure A2:**
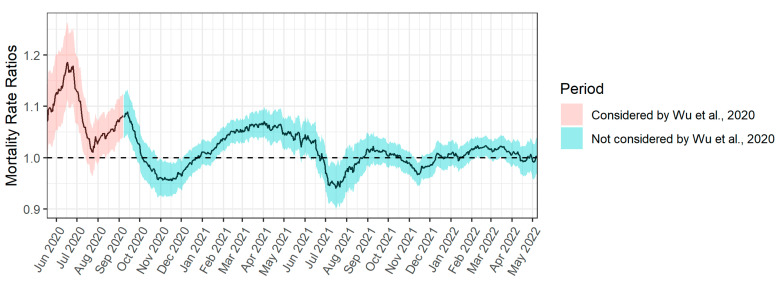
We used estimates of COVID-19 mortality from the previous 30 days as the dependent variable to recalculate the mortality rate ratios in Figure 3. For each date, we reported the 30-day lagged mortality rate ratio associated with one additional μg/m^3^ higher average long-term PM_2.5_ concentration at the county level for May 2020 through May 2022 [8]. We also shaded the 95% confidence intervals of these ratios.

## Data Availability

You can download all of the data and codes that we used from GitHub at https://github.com/bramtayl/PM_COVID_2 accessed on 10 July 2022.

## Appendix A

In Table A1, we reported the findings of many authors who studied the relationship between COVID-19 and PM_2.5_, in order of date published. We excluded studies in which authors only reported correlations. Given Figure 3, we expected to see that authors who considered data after December 2020 would find inconclusive results. Based on the date published, this does not seem to be true. Still, our findings match the findings of Tchicaya et al. in France well: “A 1 μg/m^3^ increase in the annual average PM_2.5_ concentration was associated with a statistically significant increase in the COVID-19 mortality rate, corresponding to 24.4%, 25.8%, 26.4%, 26.7%, 27.1%, 25.8%, and 15.1% in May, June, July, August, September, October, and November, respectively. This association was no longer significant on 1 and 31 December 2020” [4]. Thus, a similar phenomenon seems to have simultaneously occurred in both the United States and France.

**Table A1:** We reported the findings from a number of studies of the relationship between COVID-19 and PM_2.5_, in order of date published.

Reference	Authors	Date Published	Highlight
[16]	Jiang et al., 2020	11 May 2020	“The relative risks (RRs) of PM_2.5_ for daily COVID-19 incidences were 1.036 (95% confidence interval [CI], 1.032–1.039) in Wuhan, 1.059 (95% CI, 1.046–1.072) in Xiaogan, and 1.144 (95% CI, 1.12–1.169) in Huanggang”
[5]	Adhikari and Yin	5 June 2020	“A one-unit increase in the moving average of PM_2.5_ (µg/m^3^) was associated with a 33.11% (95% CI 31.04–35.22) decrease in the daily new COVID-19 cases.”
[17]	Zhu et al., 2020	20 July 2020	“A 10-μg/m^3^ increase in PM_2.5_ was associated with a 2.24% (95% CI: 1.02 to 3.46) increase in the daily counts of confirmed cases”
[18]	Bray et al., 2020	August 2020	“There was no evidence of a significant association between PM_2.5_ and COVID-19 mortality after controlling for other variables.”
[3]	Cole et al., 2020	4 August 2020	“A municipality with 1 μg/m^3^ more PM_2.5_ concentrations will have 9.4 more COVID-19 cases, 3.0 more hospital admissions, and 2.3 more deaths.”
[19]	Coker et al., 2020	4 August 2020	“A one-unit increase in PM_2.5_ concentration (µg/m^3^) is associated with a 9% (95% confidence interval: 6–12%) increase in COVID-19 related mortality.”
[20]	Yao et al., 2020	1 November 2020	“For every 10 μg/m^3^ increase in PM_2.5_ concentrations, the COVID-19 CFR increased by 0.24% (0.01–0.48%)”
[9]	Wu et al., 2020	4 November 2020	“An increase of 1 μg/m^3^ in the long-term average PM_2.5_ is associated with a statistically significant 11% (95% CI, 6 to 17%) increase in the county’s COVID-19 mortality rate”
[21]	Stieb et al., 2020	December 2020	“Long-term PM_2.5_ exposure exhibited a positive association with COVID-19 incidence (incidence rate ratio 1.07, 95% confidence interval 0.97–1.18 per μg/m^3^)”
[22]	Travaglio et al., 2021	1 January 2021	“An increase of 1 m^3^ in the long-term average of PM_2.5_ was associated with a 12% increase in COVID-19 cases.”
[13]	Chakrabarty et al., 2021	15 March 2021	“An increase of 1 μg/m^3^ in PM_2.5_ levels below current national ambient air quality standards associates with an increase of 0.25 in R0 (95% CI: 0.048–0.447).”
[23]	De Angelis et al., 2021	April 2021	“An increase of 10 μg/m^3^ in the mean annual concentrations of PM_2.5_ over the previous years was associated with a 58% increase in COVID-19 incidence rate.”
[24]	Lorenzo et al., 2021	June 2021	“Every 1 μg/m^3^ increase (15-day MA) in PM_2.5_ was significantly associated with a 22.6% (95% CI: 12.0%–34.3%) increase in the average daily number of COVID-19 cases.”
[25]	Fang et al., 2021	10 June 2021	“Each 1-µg/m^3^ increase in annual average concentration of PM_2.5_ exposure was associated with 7.56% (95% CI: 3.76%, 11.49%) increase in COVID-19 risk.”
[15]	Mendy et al., 2021	30 August 2021	“A 1 μg/m^3^ increase in 10-year annual average PM_2.5_ was associated with 18% higher hospitalization (OR: 1.18, 95% CI: 1.11–1.26).”
[4]	Tchicaya et al., 2021	6 September 2021	“A 1 μg/m^3^ increase in the annual average PM_2.5_ concentration was associated with a statistically significant increase in the COVID-19 mortality rate, corresponding to 24.4%, 25.8%, 26.4%, 26.7%, 27.1%, 25.8%, and 15.1% in May, June, July, August, September, October, and November, respectively. This association was no longer significant on 1 and 31 December 2020.”
[6]	Milicevic et al., 2021	October 2021	“A relative change in R0, with variations in pollution levels observed in the USA, is typically ~30%”
[26]	Xu et al., 2022	1 January 2022	“With every 10 μg/m^3^ increase in mean pollutant concentration, the number of daily confirmed cases increases by 9.41% (CI: 8.77%–10.04%) for PM_2.5_”
[27]	Briz-Redón et al., 2022	6 January 2022	“No associations between either PM_2.5_ exposure or environmental conditions and COVID-19 transmission were found during the early spread of the pandemic.”

## Appendix B

In Figure A1, we used the data that Wu et al. stored in the “updated_data” branch of their GitHub repository to recalculate Figure 3. In this branch, Wu et al. included PM_2.5_ concentrations for two additional years: 2017 and 2018.

In Figure A1, the decline in the association between PM_2.5_ air pollution and COVID-19 mortality after September 2020 was even sharper. The mortality rate ratio declined sharply and never again rose above statistical significance. Using data available up until May 2022, we estimated that a 1 μg/m^3^ increase in PM_2.5_ caused a 0.603% increase in COVID-19 mortality rates, with a 95% confidence interval of between a 0.560% decrease and a 1.78% increase. This interval included zero, and the upper bound was far below the upper bound that Wu et al. estimated.

## Appendix C

In Figure A2, we used lagged estimates of mortality from the previous 30 days as the dependent variable to recalculate the mortality rate ratios in Figure 3. This allowed us to see mortality rate ratios that reflected trends over only the past month, not over all previous dates, as in Figure 3. The general pattern in this figure was similar to the general pattern in Figure 3. However, these trends in mortality rate ratios were much more varied because of the shorter time periods. Given Figure 3, one might expect that, after September 2020, monthly mortality rate ratios fell below one to balance out mortality rate ratios above one in previous months. This is not uniformly true. Instead, the effect of the early strong relationship with PM_2.5_ concentrations in early months dissipated over time as new data that did not reflect this relationship accumulated.
